# Toxicity Evaluation of Graphene Oxide in Kidneys of Sprague-Dawley Rats

**DOI:** 10.3390/ijerph13040380

**Published:** 2016-03-29

**Authors:** Anita K. Patlolla, Jonathan Randolph, S. Anitha Kumari, Paul B. Tchounwou

**Affiliations:** 1 NIH—Center for Environmental Health, College of Science Engineering and Technology, Jackson State University, Jackson, MS 39217, USA; paul.b.tchounwou@jsums.edu; 2 Department of Biology, Jackson State University, Jackson, MS 39217, USA; 3 CESTEME Program Teacher from Jackson Public School, Jackson State University, Jackson, MS 39217, USA; mustang4762@gmail.com; 4 Osmania University College for Women, Hyderabad 500001, India; anitha_shinde2001@yahoo.com

**Keywords:** graphene oxide, serum creatine, blood urea nitrogen, catalase, glutathione peroxidase, superoxide dismutase, lipid hydroperoxide, Sprague-Dawley rats

## Abstract

Recently, graphene and graphene-related materials have attracted a great deal of attention due their unique physical, chemical, and biocompatibility properties and to their applications in biotechnology and medicine. However, the reports on the potential toxicity of graphene oxide (GO) in biological systems are very few. The present study investigated the response of kidneys in male Sprague-Dawley rats following exposure to 0, 10, 20 and 40 mg/Kg GO for five days. The results showed that administration of GOs significantly increased the activities of superoxide dismutase, catalase and glutathione peroxidase in a dose-dependent manner in the kidneys compared with control group. Serum creatinine and blood urea nitrogen levels were also significantly increased in rats intoxicated with GO compared with the control group. There was a significant elevation in the levels of hydrogen peroxide and lipid hydro peroxide in GOs-treated rats compared to control animals. Histopathological evaluation showed significant morphological alterations of kidneys in GO-treated rats compared to controls. Taken together, the results of this study demonstrate that GO is nephrotoxic and its toxicity may be mediated through oxidative stress. In the present work, however, we only provided preliminary information on toxicity of GO in rats; further experimental verification and mechanistic elucidation are required before GO widely used for biomedical applications.

## 1. Introduction

Graphene and its derivatives are promising material for important biomedical applications due to their versatility. A detailed comprehensive study of the toxicity of these materials is required in context with the prospective use in biological setting. Moreover, due to the increasing application of nanotechnology, human and environmental exposures to graphene based nanomaterials are likely to increase in the future. Most recently, graphene and graphene-based nanomaterials have become popular in the industrial supermarket. As graphene technologies progress to commercialization and large-scale manufacturing, issues of material and processing safety will need to be more seriously considered. Currently, limited information about the *in vitro* and *in vivo* toxicity of graphene and its derivatives are available, and more studies are required.

Graphene oxide is a single-atomic-layered material made by the oxidation of graphite crystals, which are inexpensive and abundant. After oxidation, the hydroxyl and carboxyl groups are formed in GO and, when conjugated, such particles can be effectively dispersed in aqueous solutions [1]. It is dispersible in water, and, as a result, is easy to process. Most importantly, it can be converted back into graphene. 

Due to its large surface area, graphene oxide (GO) can be used for the immobilization of various biomolecules. GO has been proposed as a candidate for drug delivery [2,3]. Although the biological applications of GO have not been well-studied, its biocompatibility was studied successfully in fibroblast cells (L-929) [4] and it has been employed as a carrier for controlled drug delivery and the release of anticancer drugs [5,6]. Previous studies also reported that graphene-induced oxidative stress on neural pheochromocytoma-derived PC12 cells [7]. Liu *et al.* [2,3] reported PEGylatednano-GO could be used to deliver water insoluble anticancer drugs without any toxicity. Various studies have shown the antibacterial activity of graphene-based materials [8,9,10,11,12]. Wang *et al.* [13] reported that GO administration in mice induced chronic toxicity and lung granuloma death. In other studies [14,15], dose-dependent pulmonary toxicity, granulomatous lesions, pulmonary edema fibrosis and inflammatory cell infiltrations were also found after GO administration. The study of Schinwald *et al.* [16] reported a pulmonary inflammatory response in rats after BSA-capped graphene administration. The number of *in vivo* studies based on tissue distribution and excretion of graphene is gradually increasing.

The ability of nanoparticles (NPs) to induce toxicity has been attributed to their increased surface reactivity [17,18].The smaller the particles are, the more surface they have per unit mass and the more reactive they are in the cellular environment. It has also been proposed that the size of NP surface area greatly increases their ability to produce reactive oxygen species (ROS) [19,20]. The small particle size allows NPs several potential routes of entry into the body including inhalation, ingestion or injection [9,10]. Once inside, NPs may translocates into the bloodstream and result in adverse biological reactions in several organ, which are considered to be the secondary major sites of interaction [11,12]. 

The kidneys are principally responsible for the removal of metabolic waste, such as urea and ammonia. However, it is believed that other waste products and toxic substances, such as NPs (nanoparticles) could also be excreted through urine [21]. The kidneys receive blood from the renal arteries, which branch directly from the dorsal aorta. Despite their relatively small size, the kidneys receive approximately 20% of the entire cardiac output, making the organs highly susceptible to xenobiotics, such as NPs [22]. In theory, both the glomerular structures (during plasma filtration) and tubular epithelial cells may be exposed to NPs. Since the major function of the kidneys is to eliminate a variety of potentially harmful substances (including the potential excretion of NPs), these organs are extremely important targets for investigation with regard to nanoparticle exposure and hazard [23]. Owing to the increasing rate of applications in medicine and the widespread availability of GOs in the environment, its safety on the mammalian renal system should not be assumed. 

This study assesses the effects, after oral administration of GO on various antioxidant biomarkers and histopathology of kidney in the rat model. The question of the health effects of GOs is quite acute and this study brings new data in a field where the largest proportion of publications have been conducted with pulmonary models. Our study is among the few to assess renal cell responses to the graphene oxide to show their potential toxic biological responses in Sprague-Dawley rats.

## 2. Chemicals and Reagents 

Graphene oxide (40 nm diameter) was purchased from Graphene Supermarket. (Reading, MA, USA) and was dissolved in water. Xylene, ethyl alcohol, paraffin wax, hematoxylin-eosin stain, Diagnostic kits for Superoxide dismutase, Catalase, Glutathione peroxidase, Lipid peroxidation assays were purchased from Calbiochem (La, Jolla, CA, USA).Serum creatinine and blood urea nitrogen kits were purchased from BIOO Scientific (Austin, TX, USA).

### 2.1. Animal Maintenance

Healthy adult male Sprague-Dawley rats (8–10 weeks of age, with average body weight (BW) of 125 ± 2 g) were used in this study. They were obtained from Harlan-Sprague-Dawley Breeding Laboratories in Indianapolis, Indiana, USA. The rats were randomly selected and housed in polycarbonate cages (18.88 in × 7.25 in × 3.76 in) (three rats per cage) with steel wire tops and corn-cob bedding. They were maintained in a controlled atmosphere with a cyclic 12 h dark/12 h light cycle, a temperature of 22 ± 2 °C and 50%–70% humidity and also with free access to pelleted feed (oval normal diet with complete balanced nutritional value for biomedical research) and fresh tap water. The rats were allowed to acclimate for 10 days before treatment.

### 2.2. Doses of Graphene Oxide

Groups of five rats each were treated with three doses of graphene oxide (GO). GO was diluted with deionized water, and orally administered using feeding needles to the rats at the doses of 10, 20, 40 mg/Kg BW. Each rat received a total of five doses at 24 h intervals. Deionized water was used as negative control and was administered in the same manner as in the treatment groups. 

All animal experiments were performed in compliance with the national guidelines for the care and use of experimental animals. Procedures involving the animals and their care conformed to the institutional guidelines, in compliance with national and international laws and guidelines for the use of animals in biomedical research [24].

The size of GO was characterized using transmission electron microscopy (TEM). The GO was homogeneously dispersed in water. A drop of the homogeneous suspension on a copper grid with a lacey carbon film and allowed to be air-dried. Images were collected using a field emission JEOL-JEM-2100F, TEM, operating at 200 KV (JEOL, Tokyo, Japan).

**Figure 1:**
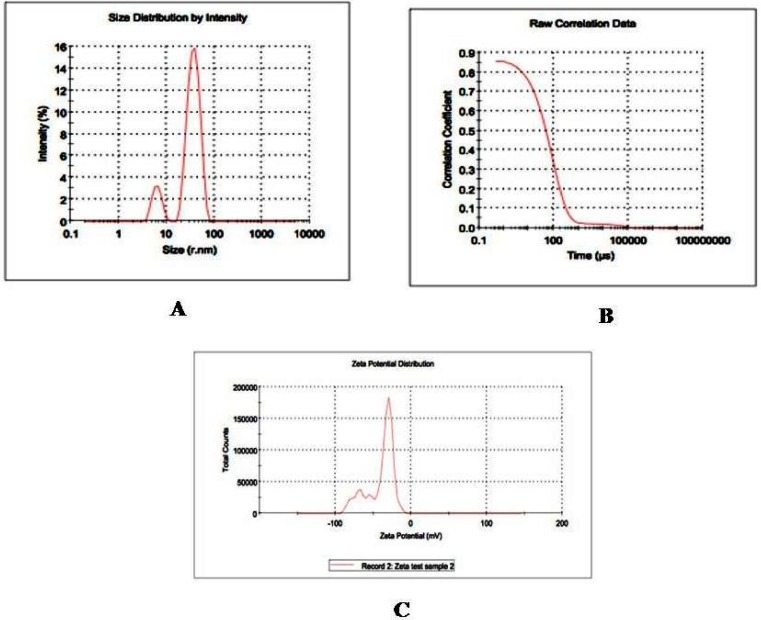
Nanostructure size and zeta potential were measured in deionized water (DI water) using a Nano Zetasizer (**A**) GOs size by Dynamic Light Scattering (DLS) intensity, (**B**) Raw correlation data; (**C**) Zeta Potential.

Nanostructure size and zeta potential were measured in deionized water (DI water) using a NanoZetasizer (Malvern, Worcestershire, UK) (Figure 1). Briefly, the nanoparticle samples were measured after dilution of a GO stock solution of 50 µg/mL in water. These dilutions were vortexed and sonicated for 5 min to provide a homogenous dispersion. For the size measurement, 1 mL of the diluted GOs dispersion was transferred to a 1 cm^2^ cuvette for dynamic size measurement. For zeta potential measurement, a Malvern zeta potential cell was washed 3–4 times with ultrapure water followed by transferring 850 µL of diluted GOs dispersion to this cell to measure the zeta potential. To assure the quality of the data the concentration of the samples and experimental methods were optimized. Sixty-nanometer NIST standard gold nanoparticles was used in the validation of the instrument. Both size and zeta potential were measured at least three times. The data was calculated as the average size or zeta potential of GOs. 

### 2.3. Preparation of Homogenates

At the end of 5 days exposure to GOs, the kidneys were excised under anesthesia. The organs were washed thoroughly in ice-cold physiological saline and weighed.Ten percent homogenate of each tissue was prepared separately in 0.05 M phosphate buffer (pH 7.4) containing 0.1 mM EDTA using a motor driven Teflon-pestle homogenizer (Fischer), followed by sonication (Branson Sonifer, Danbury, CT, USA), and centrifugation at 500× *g* for 10 min at 4 °C. The supernatant was decanted and centrifuged at 2000× *g* for 60 min at 4 °C. The cellular fraction obtained was called “homogenate” and was used for the assays.

### 2.4. Catalase (CAT) Assay

The method of Wheeler *et al.* [25] was used in analyzing the activity of catalase in kidneys of rats. Briefly, the tissues were rinsed with phosphate buffer, pH 7.4, to remove excess blood, and were homogenized (1:8, w/v) in cold buffer containing 50 mM potassium phosphate, pH 7.0 and 1 mM ethylene diamine tetra acetic acid (EDTA) per gram tissue. The supernatant from each tissue was used as the enzyme source and analyzed with 96 well plates using commercial catalase assay kit. Three replicates of 20 µL sample were mixed with 100 µL of 100 mM potassium phosphate, pH 7.0 and 30 μL methanol. The reaction was initiated with 20 µL of 35 mM hydrogen peroxide and incubated the mixture on shaker at room temperature for 20 minutes. The reaction was terminated by adding 30 µL of 10 M potassium hydroxide. Thirty microliters of 4-amino-3-hydrazino-5-mercapto-1,2,4-triazole (chromogen) was added to the three replicates of each sample then incubated on a shaker for 10 min at room temperature. After incubation, 10 µL of Calbiochem supplied potassium periodate was added to the mixture again and incubated for 5 min at room temperature. Finally, the reaction mixture’s absorbance was recorded at 540 nm using 96 well plate reader (Multiskan Ascent, Lab systems USA). The principle of the assay is based on the reaction of the catalase with methanol in the presence of an optimal concentration of H_2_O_2_ and the produced formaldehyde was measured. One unit of catalase is defined as the amount of catalase that will cause the formation of 1.0 nmol formaldehyde per min at 25 °C. A reference standard curve was prepared with formaldehyde solution.

### 2.5. Superoxide Dismutase (SOD) Assay

The method of Mattiazz *et al.* [26] was followed to analyze the activity of SOD in the kidneys of rats. The tissues were rinsed with phosphate buffer, pH 7.4, containing 0.16 mg/mL heparin, to remove excess blood. The tissues were homogenized (1:8, w/v) in cold 20 mM HEPES buffer, pH 7.2, containing 1 mM EGTA, 210 mM mannitol, and 70 mM sucrose per gram tissue. The each tissue homogenate was centrifuged at 1500× *g* for 5 min at 4 °C (Beckman XL-100K, GMI, Ramsey MN, USA). The supernatants were further analyzed with 96 well plate using commercial SOD assay kit. Three replicates of 10 µL of each sample were mixed with 200 µL of radical detector. Radical detector was prepared with 50 µL of Calbiochem supplied tetrazolium salt solution and 19.95 mL of 50 mM Tris-HCl (pH 8.0, containing 0.1 mM diethylenetriaminepentaacetic acid (DTPA) and 0.1 mM hypoxanthine). The reaction was initiated by adding 20 µL of xanthine oxidase and the reaction mixture was incubated on a shaker for 20 min at room temperature. The xanthine oxidase was prepared with 50 µL of Calbiochem supplied xanthine oxidase solution and 1.95 mL of 50 mM Tris-HCl, pH 8.0. After 20 min of incubation, the reaction mixture’s absorbance was recorded at 450 nm using 96 well plate reader (Multiskan Ascent, Lab systems USA). The assay principle is based on the utilization of a tetrazolium salt for detection of superoxide radicals generated by xanthine oxidase and hypoxanthine. One unit of SOD is defined as the amount of enzyme needed to exhibit 50% dismutation of the superoxide radical. The standard reference curve was prepared with solution of bovine erythrocyte SOD.

### 2.6. Glutathione Peroxidase (GPx) Assay

The method of Anderson *et al.* [27] was followed to analyze the activity of GPx in the kidneys of rats.The tissues were perfused with phosphate buffer saline (PBS), pH 7.4, and containing 0.16 mg/mL heparin, to remove excess blood. The tissues were homogenized (1:8,w/v) in cold homogenizing buffer contains 50 mM Tris- HCl, pH 7.5, 5 mM EDTA and 1 mM DTT per gram tissue. The homogenates were centrifuged at 10,000× *g* for 15 min at 4 °C and the each tissue supernatant was separated (Beckman XL-100K, USA). Three replicates of 20 µL of each sample was mixed with 100 µL of 50 mM Tris-HCl, pH 7.6, containing 5 mM EDTA and 50 µL of co-substrate mixture (Calbiochem supplied NADPH, glutathione, and glutathione reductase). The reaction was initiated by adding 20 µL of cumenehydroperoxide solution, the reactions mixture was mixed properly and the absorbance was recorded at 340 nm wavelength (Multiskan Ascent, Lab systems USA). The assay indirectly measures the activity of GPx by a coupled reaction with glutathione reductase. One unit of GPx is defined as the amount of enzyme that will cause the oxidation of 1.0 nmol of NADPH to NADP^+^ per minute at 25 °C.

### 2.7. Lipid Hydro Peroxides (LPO) Assay

The tissues were homogenized (1:8, w/v) in cold HPLC-grade water. Five hundred µL of the each tissue homogenate was taken in a glass test tube and equal volume of Calbiochem supplied Extract R saturated methanol was added. The mixture was vortexed for few minutes and 1 mL of cold deoxygenated chloroform was added to the sample mixture, vortexed it thoroughly. The mixture was centrifuged at 1500× *g* for 5 min at 0 °C (Beckman XL-100K, USA) and bottom chloroform layer was collected. Five hundred microliters of the bottom chloroform was mixed with 450 µL of chloroform: methanol (2:1) mixture and 50 µL of Calbiochem supplied chromogen (thiocyanate ion). Then the mixture was incubated for 5 min and the absorbance of each sample was recorded at 500 nm wavelength using spectrophotometer (2800 Unico spectrophotometer USA). This method directly measures the lipid hydro-peroxides utilizing redox reactions with ferrous ions, the produced hydroperoxides are highly unstable and react readily with ferrous ions to produce ferric ions. The produced ferric ions were detected using thiocyanate ion as chromogen. Calbiochem supplied lipid hydroperoxides solution was used as reference standard.

### 2.8. Determination of Serum Creatinineand Blood Urea Nitrogen(BUN) Level

#### 2.8.1. Serum Creatinine Assay

To determine serum creatinine, the procedure is adapted from the method of Folin and Wu, described in Hawk, Oser and Summerson, was followed. The method is based on the Jaffe reaction. Creatinine reacts with picrate ion formed in alkaline medium to develop a red-orange color. The color produced from the sample is then compared in a colorimeter at wavelength of 505 nm with that produced by a known amount of creatinine under the same condition. Ten milliliters of the 100 mM (100 nmole/mL) Creatinine Standard Solution was added to 990 mL of Creatinine Assay Buffer to prepare a 1 mM (1 nmole/mL) standard solution. 0, 2, 4, 6, 8, 10 mL of the 1 mM Creatinine standard solution was added into a 96 well plate, generating 0 (blank), 2, 4, 6, 8, and 10 n mole/well standards. Creatinine Assay Buffer was added to each well to bring the volume to 50 mL. Fifty milliliters of the appropriate Reaction Mix was added to each of the wells. It is mixed well using a horizontal shaker or by pipetting, and the reaction was incubated for 60 min at 37 °C. The plate was protected from light during the incubation. The absorbance was measured at 570 nm (A570).

Concentration of Creatinine
Sa/Sv = C
Sa = Amount of Creatinine in unknown sample (nmole) from standard curve
Sv = Sample volume (mL) added into the wells
C = Concentration of Creatinine in sample
Creatinine molecular weight: 113.12 g/mole

#### 2.8.2. Blood Urea Nitrogen Assay

The MaxDiscovery Blood Urea Nitrogen Enzymatic Kit (BIOO Scientific, Austin, TX, USA) measures the concentration of urea using the urease enzyme, which converts urea to ammonia.

$$\text{CO}\;({\text{NH}}_{2})+H_{2}O\;\underset{\to }{\text{urease}}\;{\text{CO}}_{2}+2\;{\text{NH}}_{3}$$

Is a microplate-based colorimetric assay for the determination of urea in serum samples. Blood urea nitrogen (BUN) is an important marker for normal kidney function. 0.2–1 mL blood sample were allowed to coagulate in a micro centrifuge tube for 20 min at 37 °C and then centrifuged for 5 minutes at 9000 rpm. The supernatant (serum) was transfer to a clean tube. The serum samples were diluted before testing to 1:4 in either normal saline or phosphate buffer saline (PBS) (dilution factor = 5). Five microliters of diluted serum in duplicate was added to the microplate wells; then, 150 uL of Urease Mix Solution was added to the wells. The plate was tapped gently 3–4 times to mix the sample and enzyme. It was left at room temperature to incubate for 15 min. Then, 150 uL of Alkaline Hypochlorite was added to each well and incubated for 10 min at room temperature. The absorbance of each sample in duplicate was measured at 620 nm. A calibration curve of urea standards was used to determine the urea concentration in the samples. The urea concentration (dilution factor) in the well can be determined using the equation.

Blood Urea Concentration = dilution factor X (average absorbance—y-intercept) slope. 

### 2.9. Histopathological Evaluation

Kidneys were surgically removed from rats under diethyl ether anesthesia. Portions of kidney were taken and washed with ice-cold normal saline (0.9% NaCl) and 20 mM EDTA to remove blood, cut into small pieces, and fixed immediately in 10% phosphate-buffered formalin for 48 h. The tissues were then transferred to 70% ethyl alcohol and stored until processed. The tissue specimens (kidney) were processed, embedded in paraffin, sectioned at 0.1 µm, and stained with hematoxylin and eosin (HE) for histological examination under a light microscope. The extent of tissue injury was estimated semi-quantitatively and lesions scored as multi-focal fibrosis/necrosis. At least 20 slides of each sample were scored for kidney histology. Histopathology of kidney was evaluated in terms of tubular injury defined as tubular dilation, tubular necrosis, renal tubular separation, disintegration of tubules and glomerular necrosis. Glomerular cells and all kinds of tubules were included in the scoring system: 0 = no injury, 1 ≤ 10% of injury, 2 = 10%–25% of injury, 3 = 26% to 50% of injury, 4 = 51%–75% of injury and 5 ≥ 75% of injury.

### 2.10. Statistical Analysis

Statistical analysis was performed with SAS 9.1 software for Windows XP (USA). Data were presented as Means ± SDs. One-way analysis of variance (ANOVA) with *p*-values less than 0.05 were considered as statistically significant. Dunnett’s *T*-Test was used for *post hoc* evaluation of the data.

## 3. Results

### 3.1. Nanomaterial Characterization

**Figure 2:**
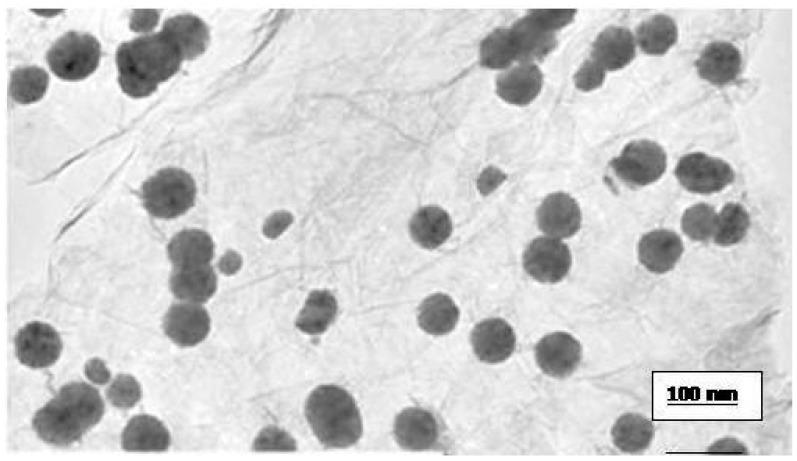
Transmission electron microscope picture of graphene oxide (GO). Diameter size: 38 nm.

Morphology, diameter, tendency of aggregation and cellular distribution of nanoparticles were characterized by using transmission electron microscope (TEM) (JEOL-1011). Mainly, spherical shaped graphene oxide (GO) was observed (Figure 2). To understand the state of dispersion of the particles when placed into deionized water (DI water), the GOs sample was analyzed by dynamic light scattering (DLS) (Figure 1). The results from DLS showed agglomeration of GOs more than its primary size (40 nm), and the zeta potential value of GOs was shown to be −33.2 mV. A solution is considered stable if the zeta potential value is more negative than −30 mV or more positive than 30 mV.

### 3.2. Biochemical Analysis

**Figure 3:**
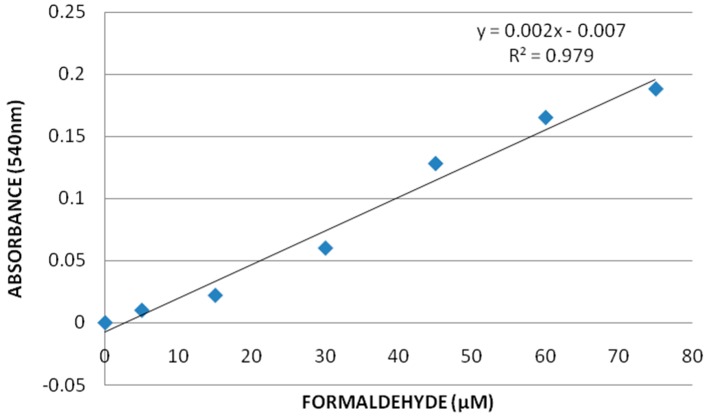
Catalase (CAT) assay standard curve where y-axis represents the net absorbance at 540 nm and x-axis represents the various concentrations of reference standards, formaldehyde (µM).

**Figure 4:**
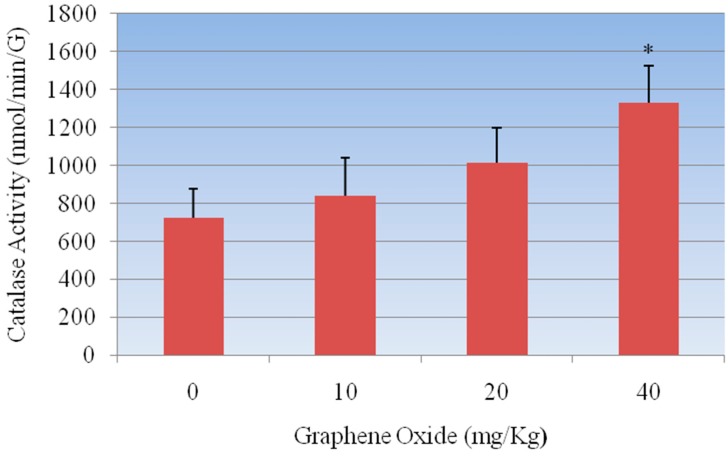
Effect of GOs on CAT activities in kidneys of rats after 5 days. Each bar represents mean ± SD of five rats. Values with asterisks were significantly different from control. Statistical significance (*p* < 0.05) is depicted as (*).

**Catalase Assay:** The standard curve of formaldehyde was used to determine the activity of catalase in rat kidney tissues, which is represented in Figure 3. The results of this assay showed an increase in the catalase activity for GO-treated groups compared to the control. The catalase activity in kidney was 723.2 ± 152.3, 839.5 ± 203, 1012.9 ± 186.1, and 1329.03 ± 17.5 nmol/min/gram tissue for 0, 10, 20, and 40 mg/Kg GO, respectively. There was a concentration-dependent increase in the catalase activity compared to control, however, statistically significant effect was observed in 40 mg/Kg GO in the catalase activity compared to control. Figure 4 represents the catalase activity data.

**Figure 5:**
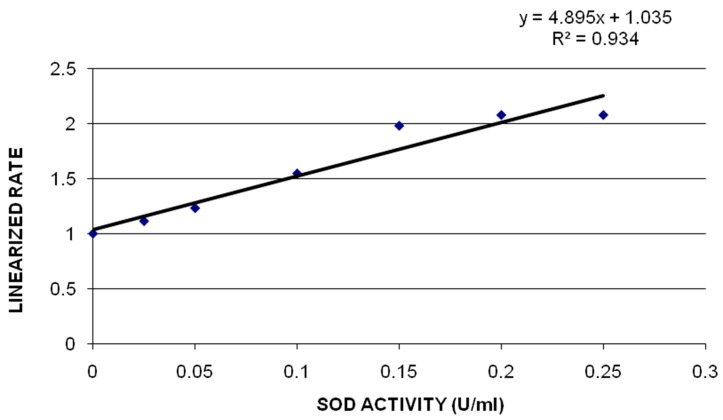
SOD standard curve where y-axis represents the linear rate of absorbance (450 nm) and x axis represents the SOD activity of reference standard, bovine erythrocyte SOD, expressed in U/ml.

**Figure 6:**
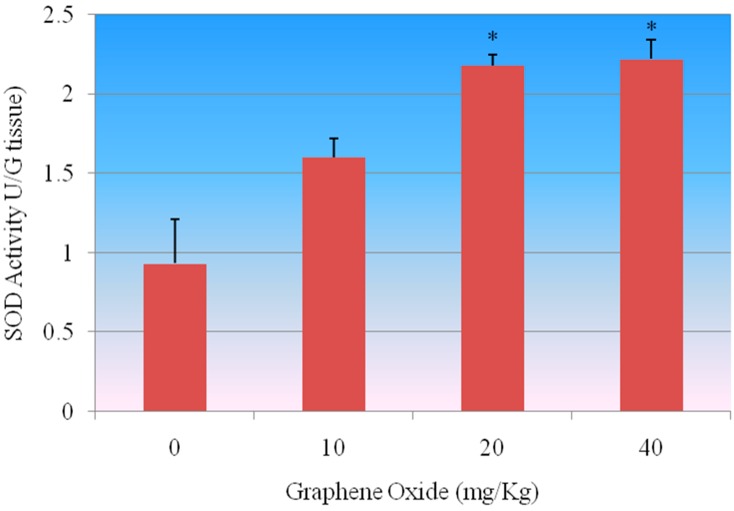
Effect of GOs on SOD activities in kidneys of rats after 5 days. Each bar represents mean ± SD of five rats. Values with asterisks were significantly different from control. Statistical significance (*p* < 0.05) is depicted as (*).

**Superoxide Dismutase (SOD) Assay:** SOD assay was performed to determine the SOD activity in kidney tissue of rats after being exposed to 0, 10, 20 and 40 mg/Kg GO. Figure 5 shows the SOD standard curve where y-axis represents the linear rate of absorbance (450 nm) and x axis represents the SOD activity of reference standard, bovine erythrocyte SOD, expressed in U/mL. The SOD activity of control and treatment groups of kidney is presented in Figure 6. The SOD activity levels in kidney were 0.932 ± 0.03, 1.6 ± 0.12, 2.18 ± 0.07 and 2.2 ± 0.12 units/gram tissue for 0, 10, 20 and 40 mg/Kg GO, respectively. SOD activity was increased with the increasing concentration of GO in treatment groups compared to the control groups. The SOD activity in kidney was significantly increased in the two highest concentrations, 20 mg/Kg and 40 mg/Kg, of GO compared to the control.

**Figure 7:**
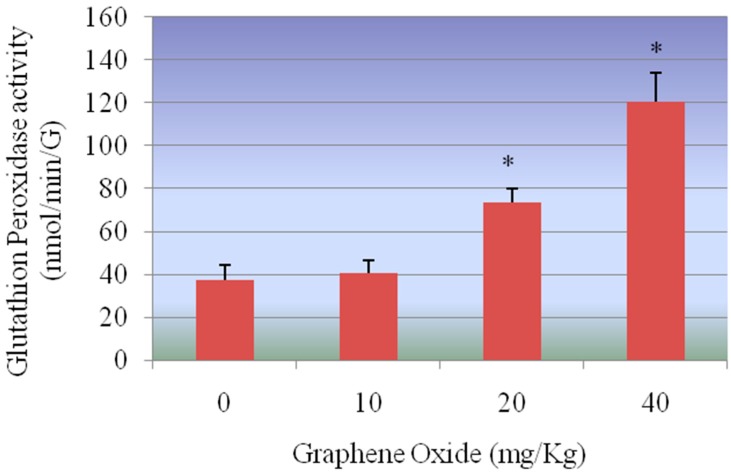
Effect of GOs on GPx activities in kidneys of rats after 5 days. Each bar represents mean ± SD of five rats. Values with asterisks were significantly different from control. Statistical significance (*p* < 0.05) is depicted as (*).

**Glutathione peroxidase (GPx) assay:** Glutathione peroxidase activity of control and treatment groups of rat kidneys are presented in Figure 7. The GPx activity levels were 37.2 ± 7.2, 40.5 ± 26.1, 73.6 ± 26.7 and 120.5 ± 13.34 nmol/min/gram tissue for 0, 10, 20 and 40 mg/Kg GO, respectively. GPx activities in kidneys were increased in all the treatment groups compared to the control group. However, the increase in activity of GPx in the kidney was increased significantly in 20 mg/Kg and 40 mg/Kg treatment groups compared to the control. 

**Figure 8:**
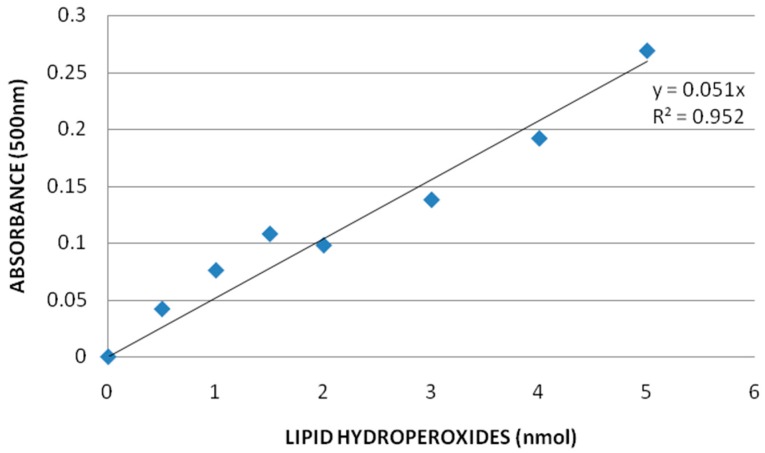
Standard curve for Lipid hydro peroxides (LPO) assay where y-axis represents absorbance at 500 nm whereas x-axis represents the concentrations of reference standard (nmol).

**Figure 9:**
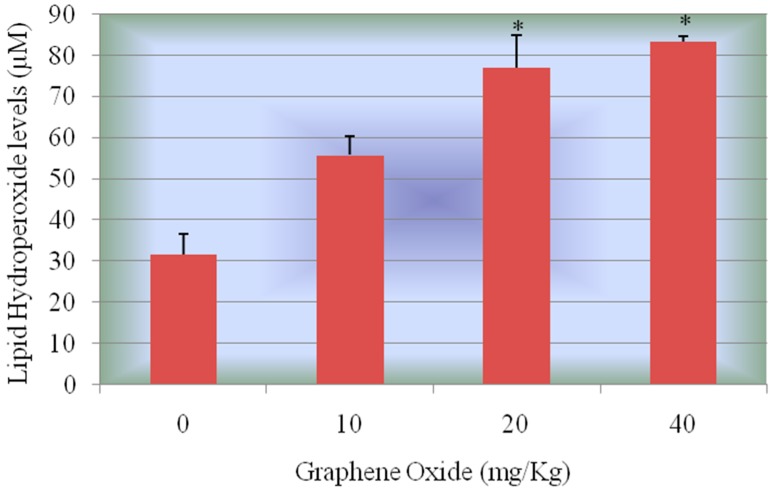
Effect of GOs on LPO activities in kidneys of rats after 5 days. Each bar represents mean ± SD of five rats. Values with asterisks were significantly different from control. Statistical significance (*p* < 0.05) is depicted as (*).

**Lipid Hydro Peroxides (LPO) Assay:** Lipid hydro peroxides assay was performed to determine the hydro peroxides levels in kidney homogenates of rats exposed to graphene oxide and controls. The LPO standard curve is presented in Figure 8. The LPO levels of the kidneys were significantly increased in all the treatment groups compared to the control group. The LPO levels in kidney were 31.7 ± 4.9, 55.78 ± 4.7, 77.1 ± 7.9, and 83.25 ± 1.3 μM for 0, 10, 20, and 40 mg/Kg GO, respectively. Figure 9 represents the experimental data of LPO.

### 3.3. Serum Creatinine

**Figure 10:**
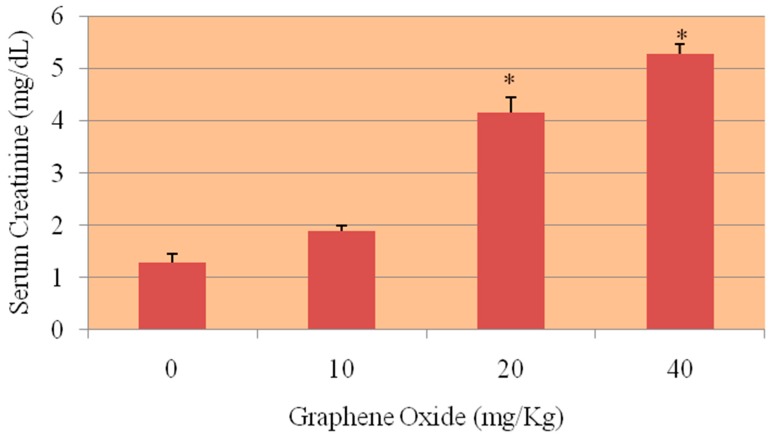
Effect of GOs on serum creatinine activities in kidneys of rats after 5 days. Each bar represents mean ± SD of five rats. Values with asterisks were significantly different from control. Statistical significance (*p* < 0.05) is depicted as (*).

Figure 10 presents the experimental data obtained from the analysis of serum creatinine. The results yielded creatinine level in serum of 1.28 ± 0.17, 1.89 ± 0.11, 4.15 ± 0.29 and 5.28 ± 0.18 for 0, 10, 20 and 40 mg/Kg of GO respectively. As shown in the figure, there was an increase in the level of creatinine in the serum of Sprague-Dawley rats. However, the highest two doses 20 and 40 mg/Kg were found to show statistically significant effect in elevating the level of creatinine when compared to control.

### 3.4. Blood Urea Nitrogen (BUN)

**Figure 11:**
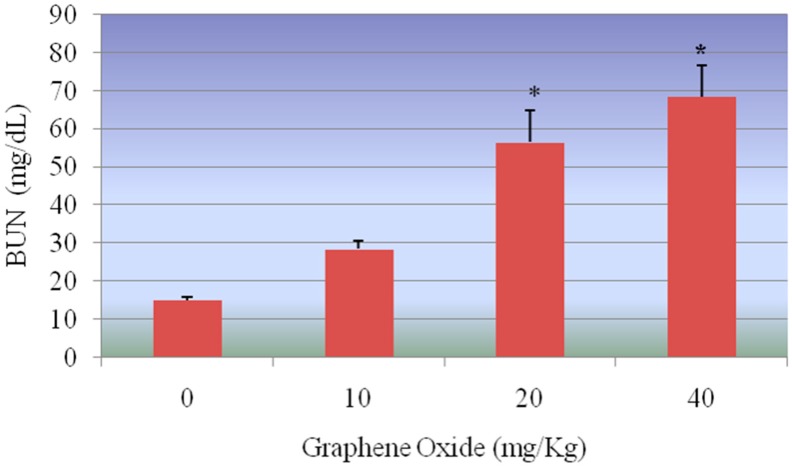
Effect of GOs on BUN activities in kidneys of rats after 5 days. Each bar represents mean ± SD of five rats. Values with asterisks were significantly different from control. Statistical significance (*p* < 0.05) is depicted as (*).

Figure 11 presents the experimental data obtained from the analysis of blood urea nitrogen (BUN). Graphene oxide (GO) exposure resulted in elevating the level of BUN in a dose-dependent manner. However, the increase was statistically significant only in the highest two doses 20 and 40 mg/Kg compared to controls. The BUN readings of 14.97 ± 0.86, 28.46 ± 2.14, 56.50 ± 8.34 and 68.32 ± 8.37 were obtained for 0, 10, 20 and 40 mg/Kg GO, respectively.

### 3.5. Histopathological Evaluation

**Figure 12:**
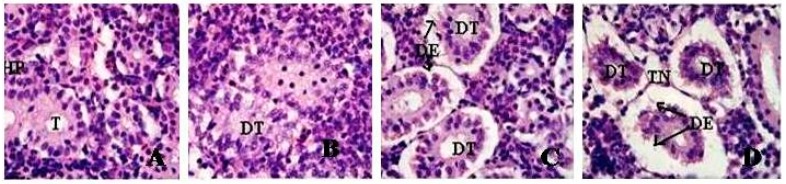
Histopathological Characterization (H&E Staining 1000 X) of Kidneys in Sprague-Dawley rats exposed to graphene oxide (GOs). (**A**) Negative Control Deionized water (T = tubule); (**B**) 10 mg/Kg exposed kidney (DT = dilated tubule, HP = Hematopoetic tissue); (**C**) 20 mg/K (DT = dilated tubule, DE = degeneration of epithelial cells); (**D**) 40 mg/Kg (DT = dilated tubule, DE = degeneration of epithelial cells, TN = Tubular necrosis).

Microscopic examination of the control rat kidney showed uniformly formed functional units called tubules and the interstices of the tubules contain hematopoietic tissue. However, the kidneys of 10, 20 and 40 mg/Kg GO exposed rats showed morphological alterations. The kidney of 10 mg/Kg exposed rats showed tubular dilation and renal tubular separation, whereas the kidneys of 20 mg/Kg exposed rats showed tubular necrosis, disintegration of tubules, degeneration of hematopoietic tissue and esonophillic exudates. Further, the kidney of rats exposed to 40 mg/Kg, progressive dilation of tubules, tubular necrosis, renal tubular separation, degeneration of hematopoietic tissue and tubular lumen were observed. Figure 12 represents the histological images of kidneys. 

## 4. Discussion

Nanoparticles/nanomaterials have been shown to enter systemic circulation; therefore, they have the potential to cause organ damage throughout the body. Those organs with extensive blood supply, such as liver, spleen, and kidneys, are especially vulnerable. The kidneys play a particularly important role as they are capable of filtering NPs out of the systemic circulation. In doing so, they are increasingly exposed to damage via those NPs they have filtered from blood. Morphological, pathological, and cellular changes from exposure to NPs that eventually lead to renal dysfunction have been studied. However, few studies have discussed the potential toxic effects of NPs on renal tubular and glomerular targets. Nanomaterials are known to affect tissue or disrupt with physiological processes by generation of reactive oxygen species (ROS) [28,29]. The antioxidant defenses comprising the enzymatic and non-enzymatic are particularly essential because they are responsible for the direct removal of free radicals, thus providing conferring protection for biological tissues, including kidneys.

The present study demonstrated that exposure of rats to GOs had adverse effects on the antioxidant status of the kidneys. The first mechanisms of antioxidant defense against the toxic effects of ROS in the tissues are carried out by SOD and CAT. The conversion of superoxide radicals to H_2_O_2_ is executed by SOD whereas the CAT subsequently decomposes H_2_O_2_ into water and oxygen. The increased SOD activity observed in the GOs-treated rats possibly indicates enzyme stimulation due to high levels of the superoxide anion radical in the kidneys of rats. Moreover, the increase in CAT and GPX activities suggests an adaptive mechanism to reduce the toxic effects of elevated levels of H_2_O_2_ in the kidneys of GOs-treated rats. An increase in CAT activity has been correlated with the overproduction of H_2_O_2_ under stress conditions [30]. The diminution in the GSH level suggests overutilization in the detoxification process so as to cope with oxidative stress. The decrease in GST activity may result from decrease substrate GSH or inhibition by increased free radicals in the kidney of GOs-treated rats. The damage due to oxidative stress in kidneys by GOs was evidenced by the elevated levels of H_2_O_2_ and LHP in the experimental rats. In this study we found that CAT, SOD, GPX activities were increased in a dose-dependent manner compared to control group, however the highest two doses 20 mg/Kg and 40 mg/Kg were found to show a statistically significant effect in increasing the activity of these antioxidants enzymes.

The current investigation also revealed that the administration of GO elevated (*p* < 0.05) serum creatinine and blood urea nitrogen (BUN) in treated groups compared to control group, with, the two highest doses of GO (20 and 40 mg/Kg) showing a statistically significant effect in elevating the serum creatinine and BUN. High levels of serum creatinine and urea levels in the blood indicate that the kidneys are not functioning properly. It could be due several factors such as kidney damage or infection, reduced blood flow to the kidneys due to shock heart failure [23].

The results of these biochemical markers were supported by the histopathological examination of kidney tissues stained with HE. Marked morphological changes in the kidney of the experimental rats were observed. The kidneys of 10 mg/Kg exposed rat showed tubular dilation and renal tubular separation, whereas the kidneys of 20 mg/Kg exposed rats showed tubular necrosis, disintegration of tubules, degeneration of hematopoietic tissue and esonophillic exudates. Further, in the kidneys of rats exposed to 40 mg/Kg progressive dilation of tubules, tubular necrosis, renal tubular separation, degeneration of hematopoietic tissue and tubular lumen were observed.

## 5. Conclusions 

In conclusion, our data showed that exposure of male rats to GOs elicited oxidative stress response in the kidneys. The toxic effects of GOs are associated with disruption of antioxidant enzyme activities, a subsequent increase in lipid peroxidation and marked morphological alteration in the experimental rats. In the present work, however, we only provided preliminary information on toxicity of GO in rats; further experimental verification and mechanistic elucidation are required before GO is widely used for biomedical applications.
